# Temporal migration patterns between natal locations of ruby-throated hummingbirds (*Archilochus colubris*) and their Gulf Coast stopover site

**DOI:** 10.1186/s40462-017-0120-2

**Published:** 2018-01-10

**Authors:** Theodore J. Zenzal, Andrea J. Contina, Jeffrey F. Kelly, Frank R. Moore

**Affiliations:** 1 Department of Biological Sciences, University of Southern Mississippi, Hattiesburg, MS 39406 USA; 2 Department of Natural Resources and Environmental Sciences, University of Illinois Urbana-Champaign, Urbana, IL 61801 USA; 3 Oklahoma Biological Survey, University of Oklahoma, Norman, OK 73019 USA

**Keywords:** Migration, Stable-hydrogen isotope ratio, Spatial patterns, Temporal patterns, Evolution, Deuterium, Gulf of Mexico, Stopover, Ruby-throated hummingbirds, Alabama

## Abstract

**Background:**

Autumn latitudinal migrations generally exhibit one of two different temporal migration patterns: type 1 where southern populations migrate south before northern populations, or type 2 where northern populations overtake southern populations *en route*. The ruby-throated hummingbird (*Archilochus colubris*) is a species with an expansive breeding range, which allows opportunities to examine variation in the timing of migration. Our objective was to determine a relationship between natal origin of ruby-throated hummingbirds and arrival at a Gulf coast stopover site; and if so, what factors, such as differences in body size across the range as well as the cost of migration, might drive such a pattern. To carry out our objectives, we captured hummingbirds at a coastal stopover site during autumn migration, at which time we collected feathers from juveniles for analysis of hydrogen stable isotopes. Using the hydrogen stable isotope gradient of precipitation across North America and published hydrogen isotope values of feathers from populations of breeding ruby-throated hummingbirds, we assigned migrants to probable natal latitudes.

**Results:**

Our results confirm that individuals from across the range (30–50° N) stopover along the Gulf of Mexico and there is a positive relationship between arrival day and latitude, suggesting a type 1 migration pattern. We also found no relationship between fuel load (proxy for migration cost) or fat-free body mass (proxy for body size) and natal latitude.

**Conclusions:**

Our results, coupled with previous work on the spatial migration patterns of hummingbirds, show a type 1 chain migration pattern. While the mechanisms we tested do not seem to influence the evolution of migratory patterns, other factors such as resource availability may play a prominent role in the evolution of this migration system.

**Electronic supplementary material:**

The online version of this article (10.1186/s40462-017-0120-2) contains supplementary material, which is available to authorized users.

## Background

The majority of forest-dwelling, avian species that breed in eastern North America migrate between temperate breeding areas and tropical wintering grounds [1]. While migration has been well studied, information is missing on the migratory patterns of many species, notably the relationship between breeding locations and arrival timing at stopover sites, areas where migrants rest and refuel while *en route* [2]. Spatiotemporal patterns of migration among populations have repercussions for resource competition at stopover sites with increasing conspecific densities [3, 4]. If populations overlap *en route*, then migrants will experience increased intraspecific competition in addition to other challenges, such as predation [5], unfamiliar habitat [6], interspecific competition [3], and weather [7].

Migratory movements involve both spatial and temporal components, which have been succinctly defined by Smith and colleagues [8]. Spatially, a species can exhibit: 1) a chain migration pattern, where northern and southern populations show the same spatial pattern on the breeding and wintering grounds; or 2) a leap-frog migration pattern, where southern breeding populations winter further north than northern breeding populations. In terms of timing, species can show: 1) a type 1 migration pattern, where southern breeding populations initiate migration prior to northern breeding populations; or 2) a type 2 migration pattern, in which northern breeding populations begin to migrate before southern breeding populations. It is possible for species to show any combination of the spatial and temporal patterns described (e.g., “type 1 chain migration”, “type 2 chain migration”, etc.; see figure 1 in [8]), due to a variety of possible mechanisms. For example, northern breeding populations may face inclement conditions earlier in the season and initiate migration before southern breeding populations. Another possible driver is seasonal resource availability, which may influence initiation of migration between regional populations. Pienkowski and colleagues [9] hypothesized two additional factors to influence migration patterns, these include intraspecific competition and the cost of migration. If regional populations exhibit differential body size, then migration patterns may have evolved to alleviate intraspecific competition between populations. Finally, the cost of migration (the amount of time and energy it takes to travel between breeding and wintering locations) can influence migration timing of regional populations.

Ruby-throated hummingbirds (*Archilochus colubris)*, hereafter “ruby-throats”, are a species with an expansive breeding range, which allows opportunities to examine variation in timing of migration. Ruby-throats are latitudinal migrants traveling between tropical wintering locations (Mexico and Central America) and temperate breeding grounds (United States and central Canada) along the same degree of longitude [10]. Preliminary analysis of spatial migration patterns in ruby-throats by Hutcheson and colleagues [11] suggests a chain migration pattern (sensu [12, 13]). Our objective is to expand on their work to examine the temporal aspect of migration (type 1 chain migration or type 2 chain migration) in ruby-throats passing through a stopover site along the northern coast of the Gulf of Mexico using stable hydrogen isotope ratios. We hypothesize that timing of rubythroats’ migration will exhibit a strong temporal relationship with distance between the breeding location and our stopover site (e.g., latitude) since their southbound migration seems tightly tied to resource availability *en route* (see [14]). Specifically, we predict that ruby-throats show a type 1 chain migration pattern given that peaks in migration phenology seem to co-occur at stopover sites in the northern and southern portions of the species range [14, 15]. We also analyze factors identified by Pienkowski and colleagues [9] that may drive temporal migration patterns. We expect individuals from northern latitudes to have higher fat-free body masses (i.e. larger body size) compared to individuals from southern latitudes. We also expect lower fuel stores from individuals originating from higher (northern) latitudes since they would have travelled a longer distance compared to more southerly individuals when arriving at our study site.

## Methods

### Field methods

We captured ruby-throats in Fort Morgan, AL (30°13′49″ N, 88°0′13″ W; see [11] for a description of the study site) between August 25 and November 1 in 2010, 2011, and 2014 using nylon mist nets (see [15] for a complete description of capture effort). Netting effort was both active (baiting some mist-nets with artificial feeders) and passive. We banded ruby-throats with a USGS aluminum band as well as sexed and aged (hatching year or after-hatching year) by bill corrugation, plumage, and morphology [16]. We estimated subcutaneous fat [17], measured natural wing chord, recorded mass, and collected two outer rectrix (R4) feathers, one from each side of the tail. We stored feather samples individually in sealed and labeled paper envelopes until analysis of stable hydrogen isotope ratios.

### Feather sampling and stable isotope analysis

For each year, we randomly selected juvenile (hatching year) individuals with feather samples (*n* = 25 for each sex per year; *n* = 150 total) throughout the autumn migration season to relate natal origin with date of passage. The feathers of hatching year birds during this phase of the annual cycle should reflect the stable hydrogen isotope ratios of the natal locations in which they were grown. We sent raw feathers to the Colorado Plateau Stable Isotope Laboratory (CPSIL; Flagstaff, Arizona, USA) for preparation and analysis of stable hydrogen isotope ratios. To prepare feathers for isotope analysis, CPSIL first cleaned the feathers with a phosphate-free detergent as well as a 2:1 chloroform methanol solution and rinsed them with deionized water before drying feathers at 50 °C overnight. CPSIL placed clipped feather material (0.350 mg; range: 0.330–0.370 mg) into silver capsules (3.5 × 5 mm) for analysis. CPSIL conducted sample pyrolysis with a Thermo Scientific TC/EA and hydrogen analysis via a Thermo Scientific Delta Plus IRMS configured through a Thermo Scientific CONFLO IV for automated continuous-flow analysis. The three normalization standards analyzed with feather samples included powdered forms of Keratin (SC Lot SJ; mean ± SD: −120.1 ± 1.0 ‰; expected: −121.6 ‰; *n* = 25), CBS – caribou hoof (mean ± SD: −197.5 ± 1.0 ‰; expected: −197.0 ‰; *n* = 8), and KHS – kudo horn (mean ± SD: −56.1 ± 1.6 ‰; expected: −54.1 ‰; *n* = 3). All δ^2^H values are reported in parts per mil (‰).

### North America isotope precipitation model

We developed a δ^2^H isoscape model based on North American precipitation values analyzed in IsoMAP ([18, 19]; see [20] for results). We considered an extensive temporal range (1980–2009) in relation to four summer months (May–August) and based our δ^2^H isoscape inferences on the following independent variables: temperature, elevation, latitude and longitude [21, 22]. For the variable “temperature”, we used min, max, and average temperatures provided by Climate Research Unit [22]. We limited the geographical extent of the δ^2^H isoscape model to a spatial range compatible with the Ruby-throated Hummingbird breeding distribution [23]. This set of variables and parameters did not show significant spatial autocorrelation necessary for developing a δ^2^H geostatistical model of reference. Therefore, we adopted the δ^2^H statistical model, which is based on multiple linear regressions between δ^2^H precipitation values and the independent variables mentioned above [24].

### Feather assignment

To determine the natal origins of migrants stopping over along coastal Alabama, we built the species feather δ^2^H isoscape using a known-origin calibration dataset of published ruby-throat feather isotope data (*n* = 186) obtained from ten breeding populations [11, 25]. Then, we used the previously generated precipitation isoscape to extract environmental δ^2^H values at the same sampling locations reported by Hutcheson and colleagues ([26, 27]; see Additional file 1: Table S1). We resampled feather and precipitation *δ*^2^H values 1000 times using the site isotopic mean and SD values of the calibration dataset to create the rescaling function from our known-origin data. We used the mean and standard deviation of these bootstrapped regressions to convert the precipitation *δ*^2^H isoscape to feather *δ*^2^H values prior to assignment (see below and “*rescale function*” and “*raster conversion function”* in Additional file 2).

We adopted a likelihood approach to obtain natal location assignment probability surfaces for each individual sampled (*n* = 150) at Fort Morgan, AL in 2010, 2011, and 2014 and to generate the corresponding assignment map for each migrant using their *δ*^2^H feather values. This allowed us to obtain the individual assignment probabilities, which virtually connected each migratory ruby-throat to its putative natal site. Our assignment computations included the tissue-specific δ^2^H values from the raster conversion step, as well as the SD values for the calibration raster and for the original raster (regression model) computed in IsoMAP (for the computation details, see [24] and Additional file 2). We normalized the assignment probabilities with the *cellStats* function in the R package “raster” [28] and constrained the probability surfaces to the Ruby-throat breeding range (see Additional file 3: Figure S1, Additional file 4: Figure S2, Additional file 5: Figure S3, Additional file 6: Figure S4, Additional file 7: Figure S5 and Additional file 8: Figure S6) using species distribution data [23].

### Assignment probabilities and latitudinal correlations

**Fig. 1:**
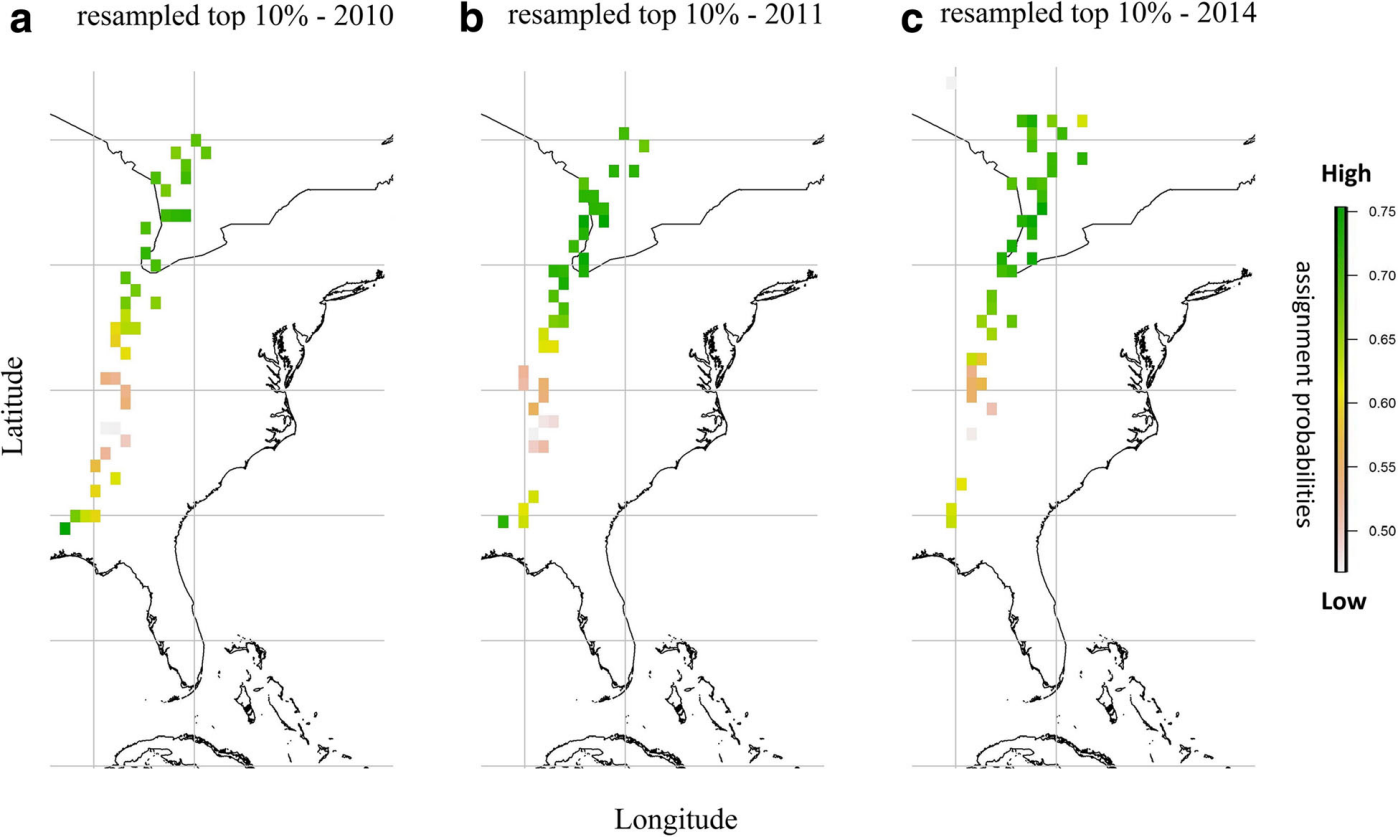
Summarized trends in natal population assignment probability surfaces among years. Each square represents the mean centroid of the top 10% of highest probabilities of predicted latitude for an individual. Color legend: dark green means high assignment probabilities, light brown means low assignment probabilities. **a** 2010, **b** 2011, **c** 2014

To summarize the origin trends among years, we explored the correlations between the inferred assignment probabilities and correspondent latitudes. Once we obtained a range of predicted natal latitudes for migrants sampled at Ft. Morgan, AL, we selected the highest probabilities within the top 10% of our dataset, which we then randomly resampled to retain only 100 values for each bird. We then used this matrix of 100 data points containing latitude, longitude, and normalized assignment probability values for each migrant to calculate the spatial centroid (i.e. geographic mean; Fig. 1) associated with the highest resampled assignment probability mean value.

### Statistical analysis

We implemented a Generalized Additive Mixed Model to investigate the linear or non-linear relationship(s) among predicted natal latitude (response variable) and non-correlated (Spearman’s rho: < 0.06) predictor variables, which included arrival day, fat-free body mass, and fuel load; year was included as a random factor. We assumed birds arrived on the date of first capture and transformed calendar date to an ordinal day. We calculated the sex-specific fat-free body mass of each individual using the data and regression method presented by Zenzal and Moore [15] for ruby-throats at Fort Morgan, AL, which is based the methods of Ellegren [29], as well as Owen and Moore [30]. The fat-free body mass of each individual allowed us to 1) estimate a proxy for each individual’s body size based on fat-free mass, and 2) estimate the fuel load of each individual by subtracting the fat-free mass from the mass at capture. We conducted our analysis using the “mgcv” package [25, 31, 32] in the R statistical language (version 3.3.3; [33]).

## Results

**Fig. 2:**
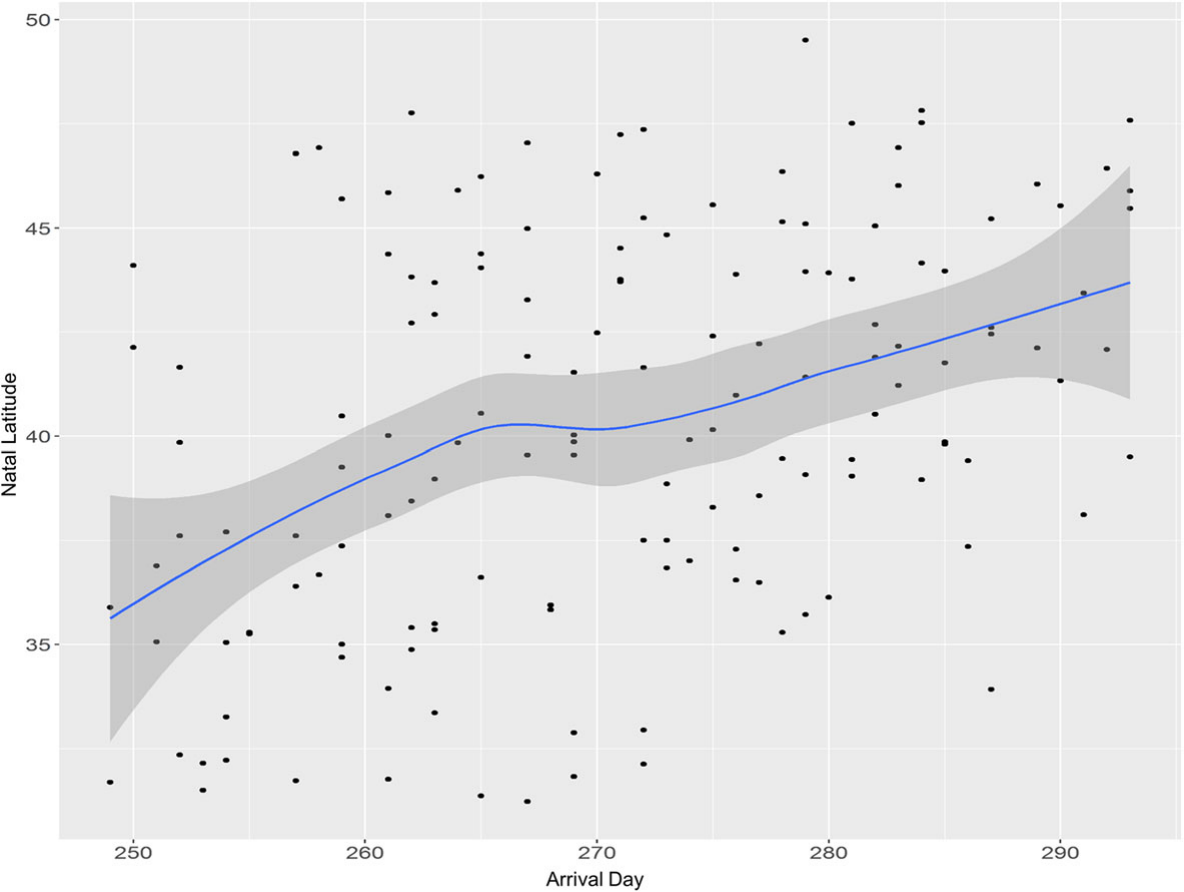
Relationship between arrival day at a Gulf coast stopover site and natal latitude. Data based on hydrogen isotope ratios of ruby-throated hummingbird tail feathers (*n* = 150). Loess regression lines are plotted with 95% confidence intervals

Our results suggest that ruby-throats passing through our study site originate from across the breeding range – the Gulf coast states through Canada (Fig. 1; Additional file 3: Figure S1, Additional file 4: Figure S2, Additional file 5: Figure S3, Additional file 6: Figure S4, Additional file 7: Figure S5 and Additional file 8: Figure S6). Arrival day and natal latitude showed a significant, positive relationship (*p* < 0.001; F_1,1_ = 28.09; Fig. 2); the direction of the relationship conforms to the type 1 chain migration, that birds originating from southern latitudes passed through our site first followed by individuals from more northern latitudes (sensu [8]). There was a weak, negative trend, which was not statistically significant, between fuel load and natal latitude (*p* = 0.10; F_1,1_ = 2.81), suggesting that there is no overall negative effect of migratory distance on fuel load at arrival. We found no pattern between natal latitude and lean body mass, a proxy for body size, indicating no difference in body size across the range (*p* = 0.58; F_1,1_ = 0.31).

## Discussion

As expected based on peaks in phenology occurring simultaneously at northern and southern stopover sites [14, 15], our results support a type 1 temporal migration pattern in juvenile ruby-throats – individuals from southern latitudes initiate migration before individuals from northern latitudes. This temporal migration pattern has also been found in sharp-shinned hawks [8] and two species of wood warbler (Parulidae; [2]) in the western United States and Canada during autumn migration. In our system, individuals from the lower-southeast (~ 30–35° N) passed through our study site in September followed by most individuals originating from higher latitudes (~ 40–50° N) in October. If we assume the same temporal pattern is exhibited as animals arrive on the wintering grounds and take into consideration previous work on the connectivity between breeding and wintering locations [26], then it is likely ruby-throats use a type 1 chain migration pattern during autumn (see figure 1a in [8]).

There are several mechanisms that have been hypothesized to drive the evolution of spatiotemporal migration patterns and we tested two purported by Pienkowski and colleagues [9]. First, we found no relationship between ruby-throat body size (fat-free body mass) and natal origin, which does not support the hypothesis that differences in body size across the range reduce intraspecific competition. This finding is similar to that of American redstarts (*Setophaga ruticilla*; [34]) and contrasts with that of sharp-shinned hawks, which show a similar temporal migration pattern [8]. However, Zenzal and Moore [15] found two distinct peaks in the phenology of juvenile ruby-throats, one peak in September and a separate peak in October, that coincidentally match when individuals from northern and southern latitudes passed through our study site as revealed by isotope analysis in this study. It is possible these peaks represent a southern population and northern population using stopover habitats along the northern coast of the Gulf of Mexico at different times, a strategy that would alleviate intraspecific competition. However, more study is needed to determine the origin of birds arriving during these separate peaks.

The second factor we tested, fuel load, did not support our prediction that there would be differences in the cost of migration across the range, rather individuals from both northern and southern latitudes arrived with similar fuel loads. These results are similar to other studies [8, 35–37] that found no relationship between fuel load and migratory distance. Moreover, most individuals in this study, as well as ruby-throats from this location previously described by Zenzal and Moore [15], typically arrived with some fuel stores. This may be due to birds being in close proximity to an ecological feature (Gulf of Mexico), where migrants tend to put on substantial fuel stores prior to negotiating a crossing (e.g., [38]). It is also possible given their small size [10] and high metabolism [39], ruby-throats always carry additional fuel stores to safeguard against times of energy shortfalls. Additionally, northern populations show synchrony between migratory movements and resource availability allowing birds to arrive on the Gulf coast in good energetic condition [14].

While beyond the scope of our study, two other mechanisms may influence migratory patterns. One possibility is that individuals show constant breeding area resident time (sensu [40]), allowing southern birds to breed earlier and hence migrate sooner than birds from northern latitudes. Another possibility is the availability of resources *en route,* which may influence when species time their migratory movements (e.g., [14, 41–44]) and this is strongly supported in hummingbirds [14, 41–43]. The timing of individuals from northern latitudes in our study tightly corresponded to the availability of *Impatiens capensis* and *Lobelia cardinalis* described by Bertin [14]. The evolution of migration patterns may be tied to the ability to obtain resources *en route,* which has important conservation and habitat management implications. While ruby-throats are not a species of conservation concern, information on timing can be used to inform the general public when to focus on providing supplemental feeding – an activity with positive economic impacts [45, 46] and likely positive impacts on survival during migration.

## Conclusions

Our results reveal that ruby-throats from across the breeding range used our coastal Alabama stopover site, with individuals from northern and southern latitudes arriving at different periods over the migratory season. When coupled with the spatial pattern described on the wintering grounds [26], ruby-throats show a type 1 chain migration pattern wherein individuals from southern latitudes initiate migration before individuals from northern latitudes. However, we were unable to identify the mechanism that might drive this pattern. We found no relationship between body size or migration cost and natal latitude, indicating other factors such as resource availability, competition, or constant breeding area resident time may be responsible for this migration pattern. Future research should work to find the mechanism(s) that may have influenced the evolution of the type 1 migration pattern in ruby-throats. 

## Additional files

Additional file 1: Table S1. Tissue and environmental δ^2^H values used for the isoscape calibration step. Supplementary literature cited. (DOCX 14 kb)

Additional file 2: R code for assignment probability. R code for the natal latitude probability assignment. (TXT 6 kb)

Additional file 3: Figure S1. Natal population assignment probability surfaces for each sampled female, hatch-year ruby-throated hummingbird captured in 2010. Feathers were collected in Fort Morgan, Alabama, USA during autumn. (PDF 1313 kb)

Additional file 4: Figure S2. Natal population assignment probability surfaces for each sampled male, hatch-year ruby-throated hummingbird captured in 2010. Feathers were collected in Fort Morgan, Alabama, USA during autumn. (PDF 804 kb)

Additional file 5: Figure S3. Natal population assignment probability surfaces for each sampled female, hatch-year ruby-throated hummingbird captured in 2011. Feathers were collected in Fort Morgan, Alabama, USA during autumn. (PDF 830 kb)

Additional file 6: Figure S4. Natal population assignment probability surfaces for each sampled male, hatch-year ruby-throated hummingbird captured in 2011. Feathers were collected in Fort Morgan, Alabama, USA during autumn. (PDF 909 kb)

Additional file 7: Figure S5. Natal population assignment probability surfaces for each sampled female, hatch-year ruby-throated hummingbird captured in 2014. Feathers were collected in Fort Morgan, Alabama, USA during autumn. (PDF 906 kb)

Additional file 8: Figure S6. Natal population assignment probability surfaces for each sampled male, hatch-year ruby-throated hummingbird captured in 2014. Feathers were collected in Fort Morgan, Alabama, USA during autumn. (PDF 752 kb)
