# Supplementary material for: Temporal migration patterns between natal locations of ruby-throated hummingbirds (Archilochus colubris) and their Gulf Coast stopover site
Source: Mov Ecol. 2018 Jan 10;6:2. doi: 10.1186/s40462-017-0120-2 (PMC5761100; doi:10.1186/s40462-017-0120-2)
Supplement: Supplementary file 1 — Tissue and environmental δ2H values used for the isoscape calibration step. Supplementary literature cited. (DOCX 14 kb) [file 40462_2017_120_MOESM1_ESM.docx]

Table S1 Tissue and environmental δ²H values used for the isoscape calibration step. Values are modified from [1,2].

| State | Sample size | Tissue | Precipitation |
| --- | --- | --- | --- |
| Alabama | 20 | -50.5 ± 7.4 | -41.0 ± 0.5 |
| Illinois | 21 | -61.9 ± 2.8 | -35.5 ± 3.3 |
| Louisiana | 21 | -46.9 ± 8.9 | -42.6 ± 0.2 |
| Michigan | 19 | -82.7 ± 6.2 | -38.7 ± 0.5 |
| Minnesota | 10 | -100.6 ± 4.9 | -52.4 ± 2.8 |
| Missouri | 10 | -62.7 ± 4.9 | -47.6 ± 2.3 |
| North Carolina | 20 | -52.4 ± 5.2 | -38.6 ± 2.1 |
| New York | 27 | -84.1 ± 10.2 | -44.7 ± 7.0 |
| Oklahoma | 19 | -58.6 ± 6.9 | -51.1 ± 10.5 |
| Pennsylvania | 19 | -76.2 ± 5.7 | -42.7 ± 6.5 |

Literature Cited for Supplementary Table

1. Hutcheson CA, Wassenaar LI, Hendrix L. A Preliminary Examination of the Use of Hydrogen Isotope Ratios in Estimating the Natal Latitudes of Hatching-Year Ruby-throated Hummingbirds. North Am. Bird Bander. 2007;32.

2. Hutcheson CA, Hendrix L, Moran JA. An isotopic analysis of migratory connectivity in ruby-throated hummingbirds. North Am. Bird Bander. 2010;35:5–11.
