# Supplementary figures and images for: Temporal migration patterns between natal locations of ruby-throated hummingbirds (Archilochus colubris) and their Gulf Coast stopover site
Source: Mov Ecol. 2018 Jan 10;6:2. doi: 10.1186/s40462-017-0120-2 (PMC5761100; doi:10.1186/s40462-017-0120-2)

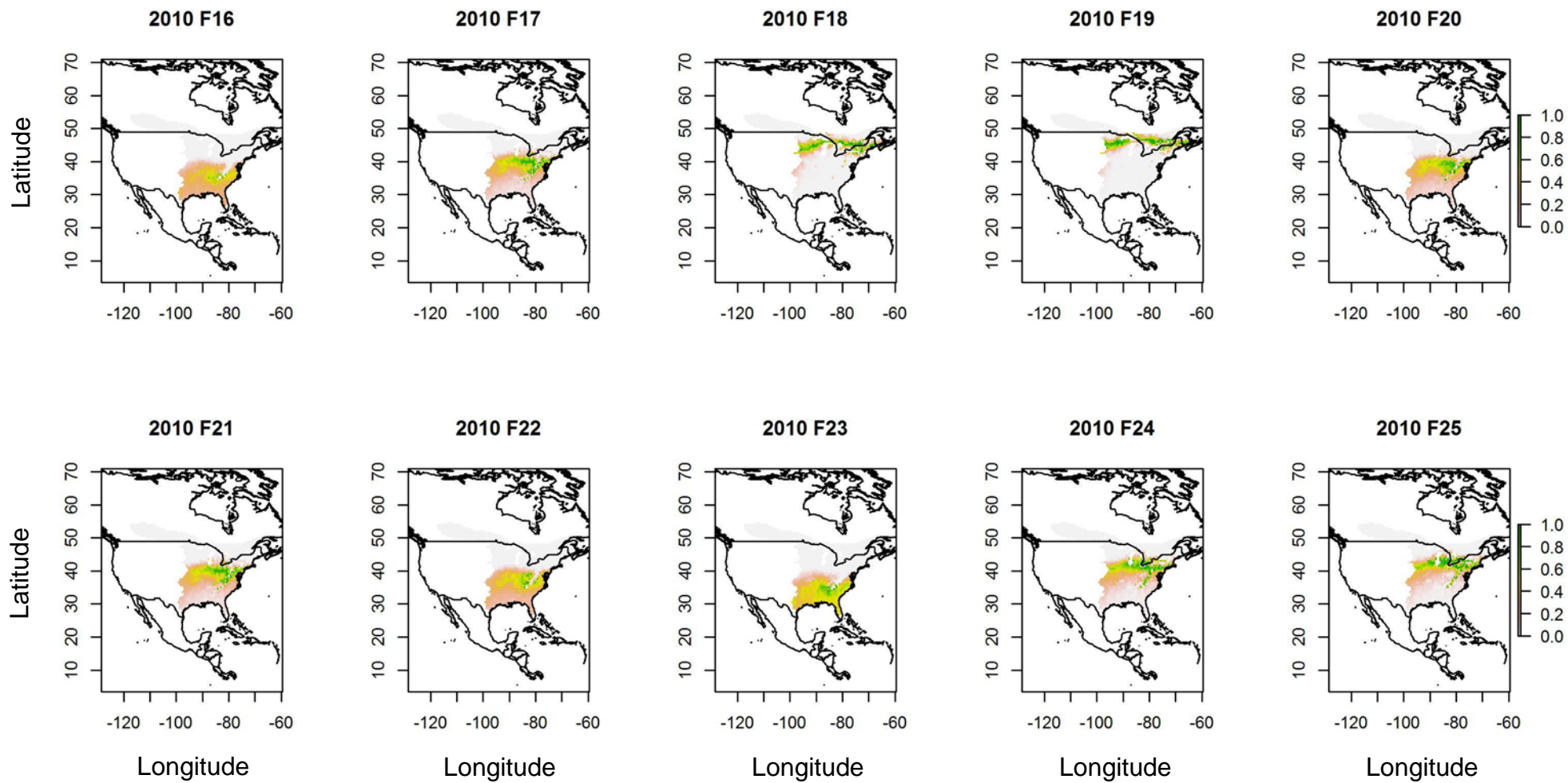

Supplement: Supplementary file 3 — Natal population assignment probability surfaces for each sampled female, hatch-year ruby-throated hummingbird captured in 2010. Feathers were collected in Fort Morgan, Alabama, USA during autumn. (PDF 1313 kb) [file 40462_2017_120_MOESM3_ESM.pdf]

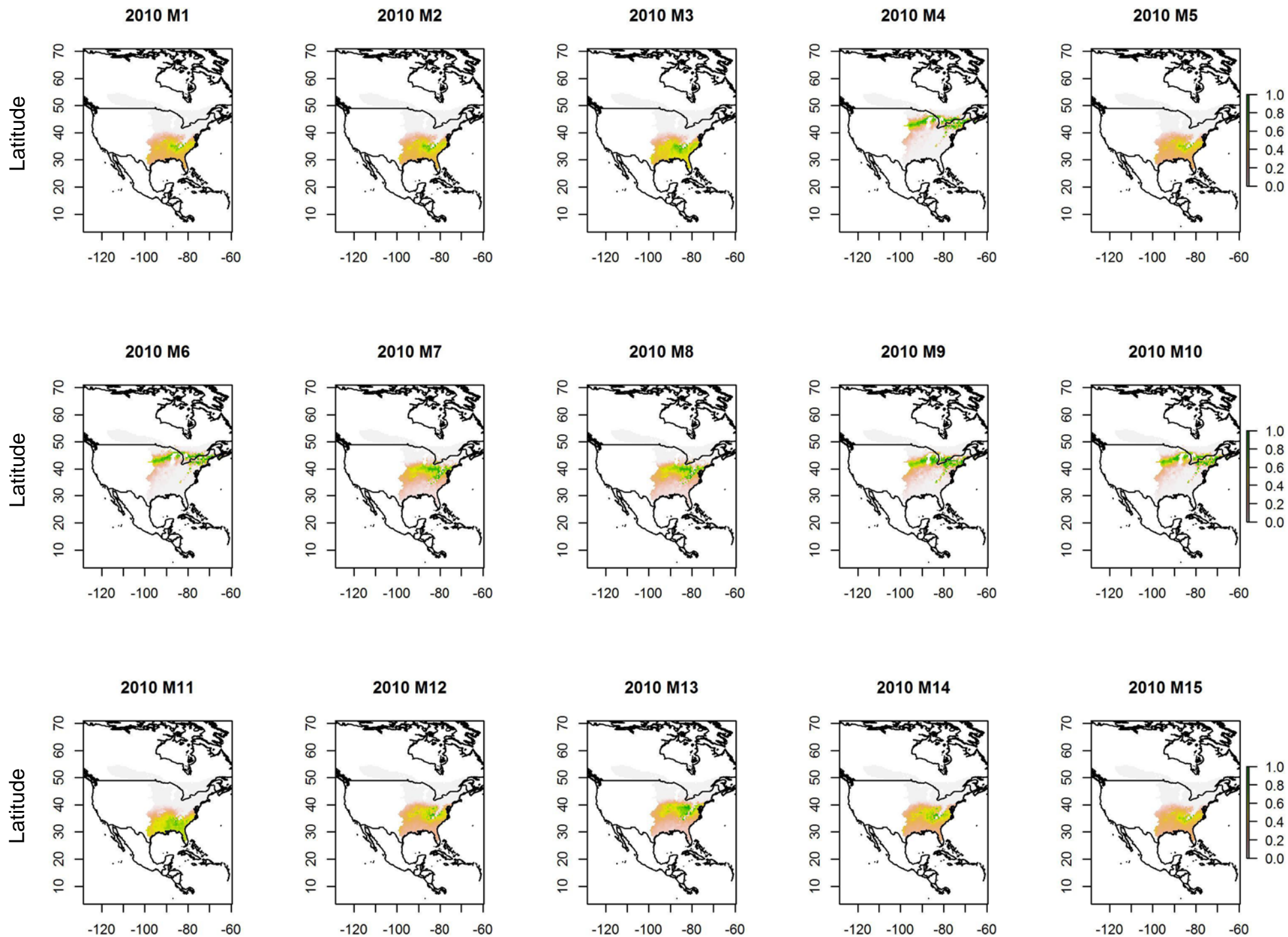

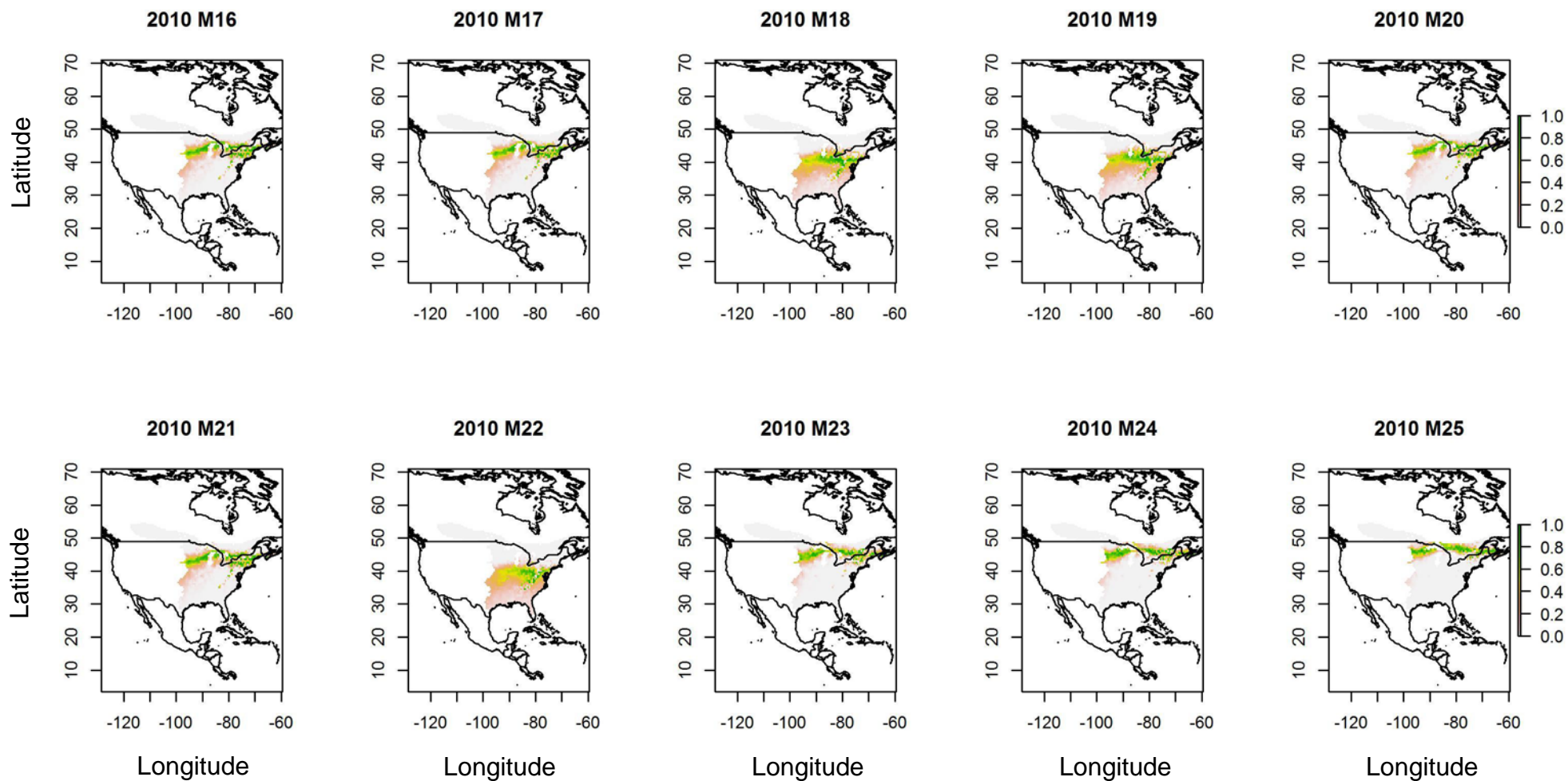

Supplement: Supplementary file 4 — Natal population assignment probability surfaces for each sampled male, hatch-year ruby-throated hummingbird captured in 2010. Feathers were collected in Fort Morgan, Alabama, USA during autumn. (PDF 804 kb) [file 40462_2017_120_MOESM4_ESM.pdf]

2011 F1

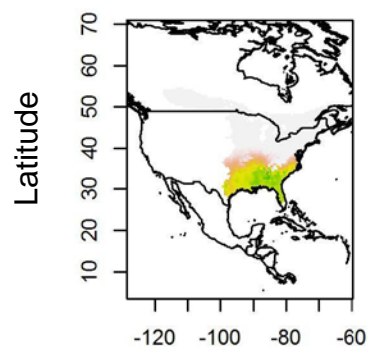

2011 F2

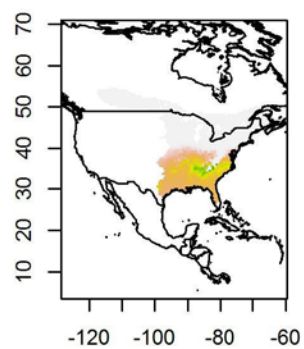

2011 F3

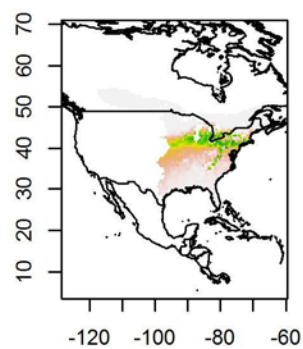

2011 F4

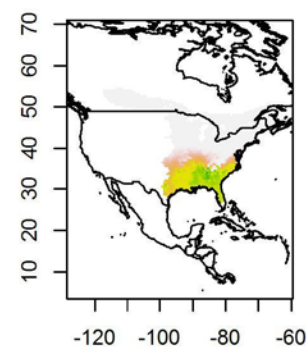

2011 F5

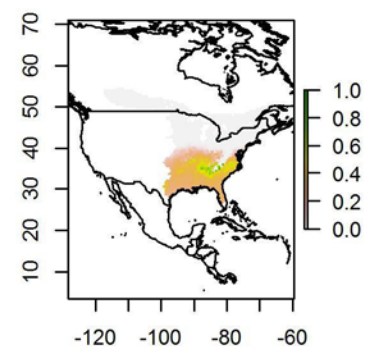

2011 F6

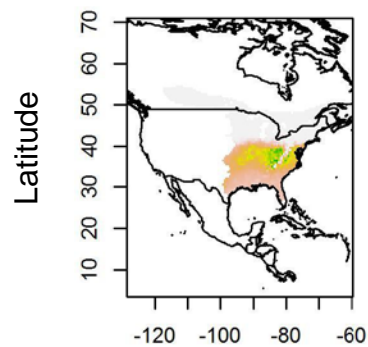

2011 F7

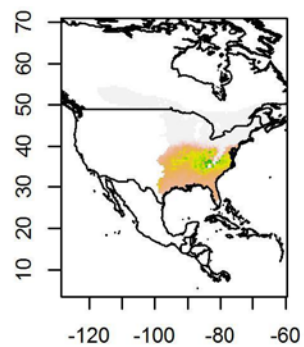

2011 F8

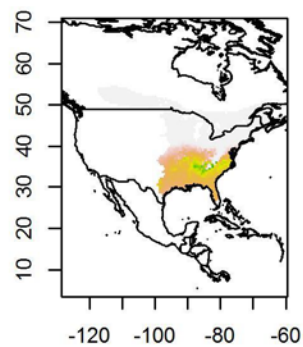

2011 F9

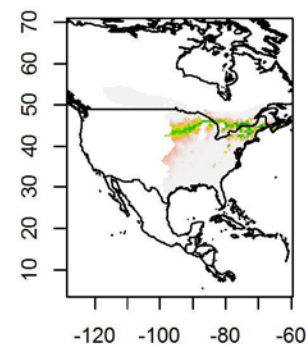

2011 F10

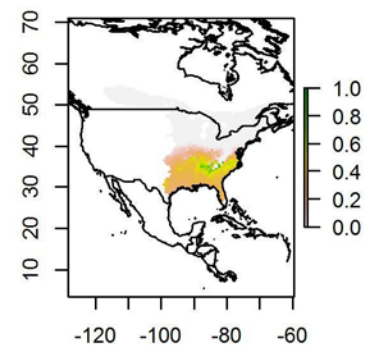

2011 F11

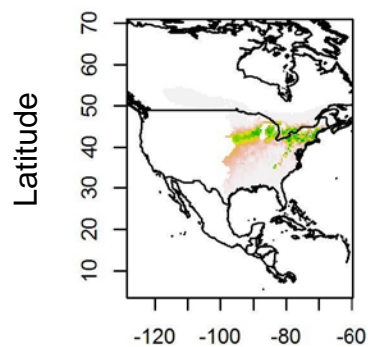

2011 F12

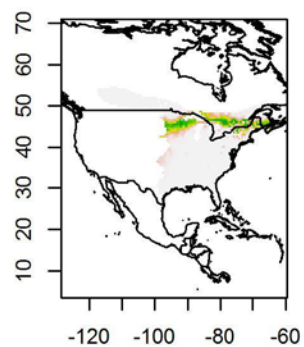

2011 F13

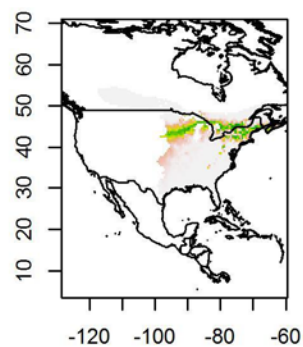

2011 F14

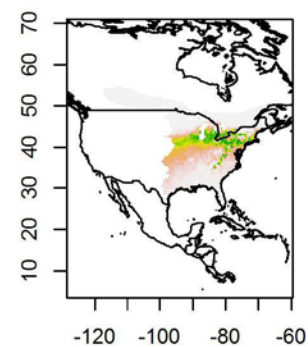

2011 F15

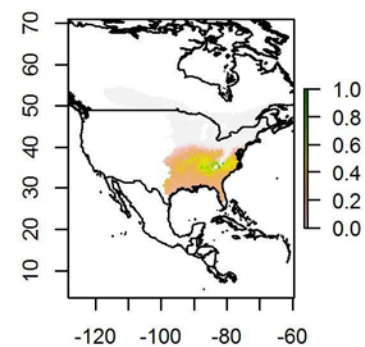

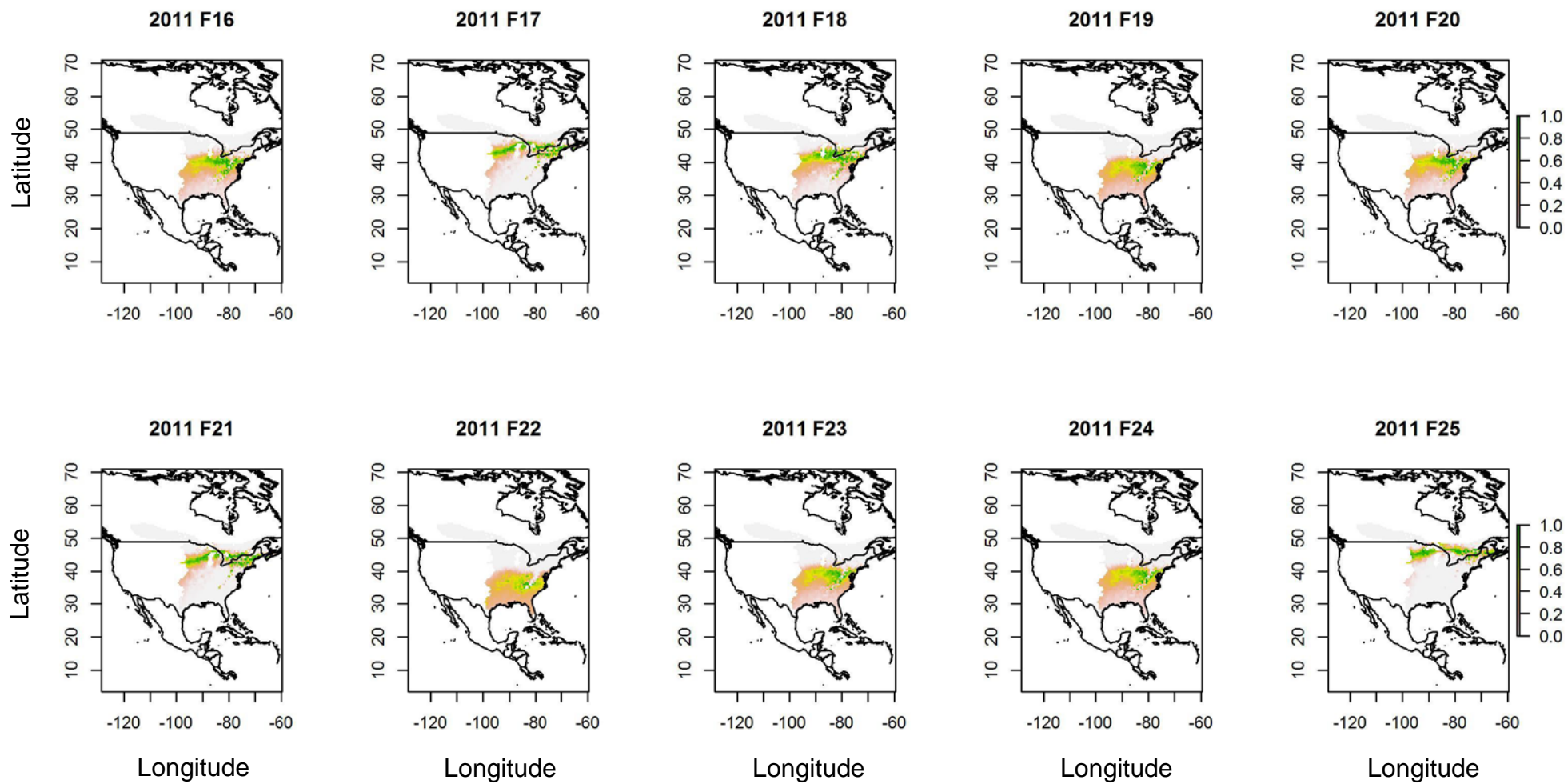

Supplement: Supplementary file 5 — Natal population assignment probability surfaces for each sampled female, hatch-year ruby-throated hummingbird captured in 2011. Feathers were collected in Fort Morgan, Alabama, USA during autumn. (PDF 830 kb) [file 40462_2017_120_MOESM5_ESM.pdf]

2011 M1

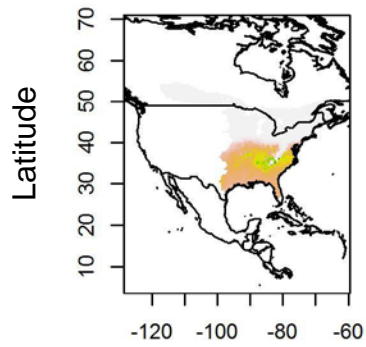

2011 M2

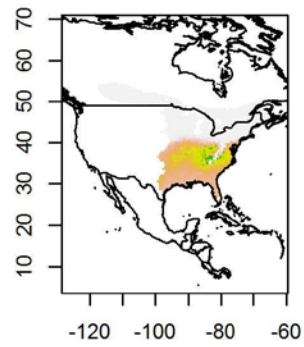

2011 M3

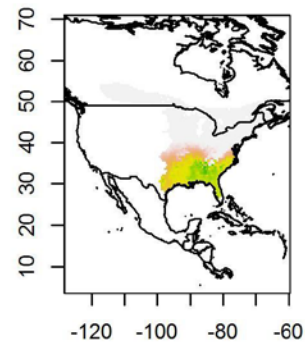

2011 M4

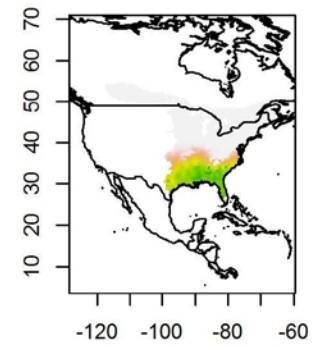

2011 M5

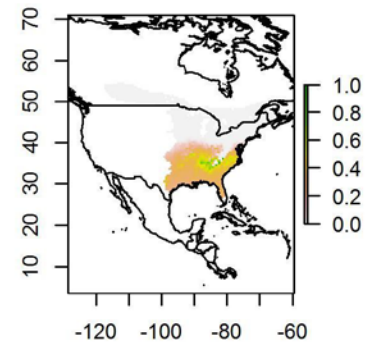

2011 M6

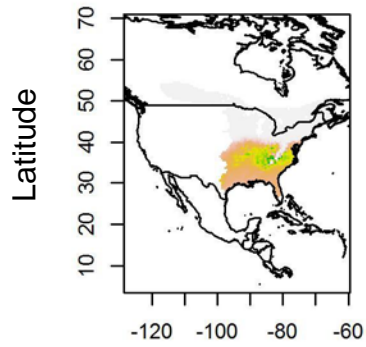

**2011 M7**

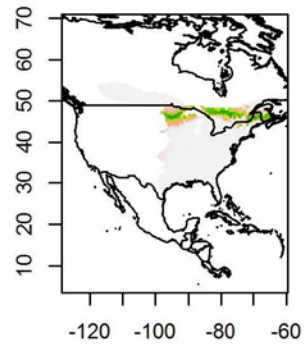

2011 M8

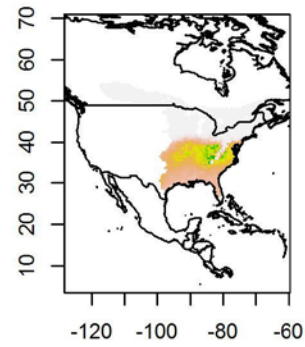

2011 M9

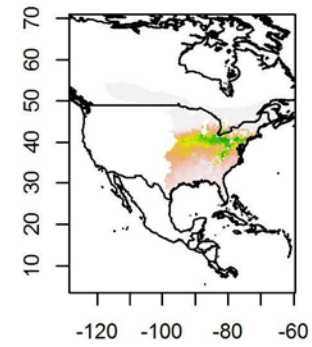

2011 M10

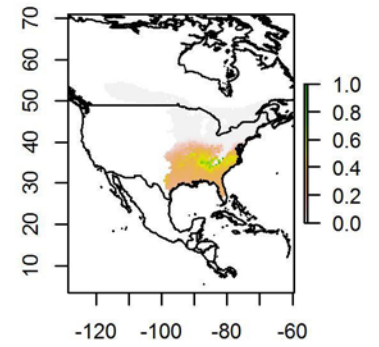

2011 M11

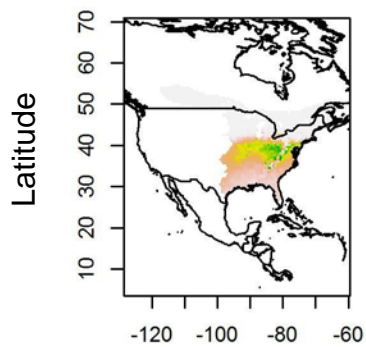

2011 M12

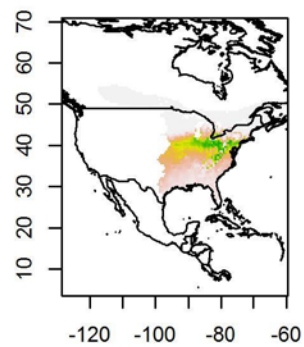

2011 M13

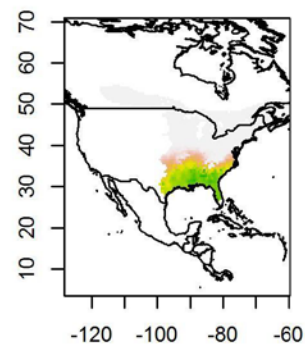

2011 M14

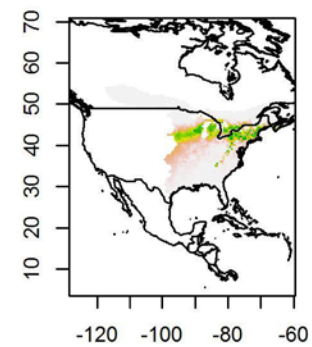

2011 M15

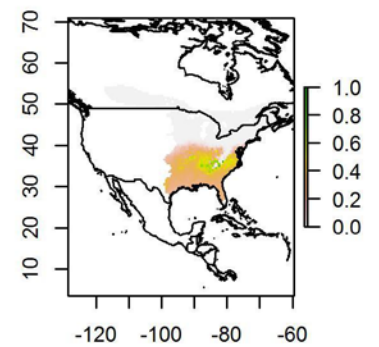

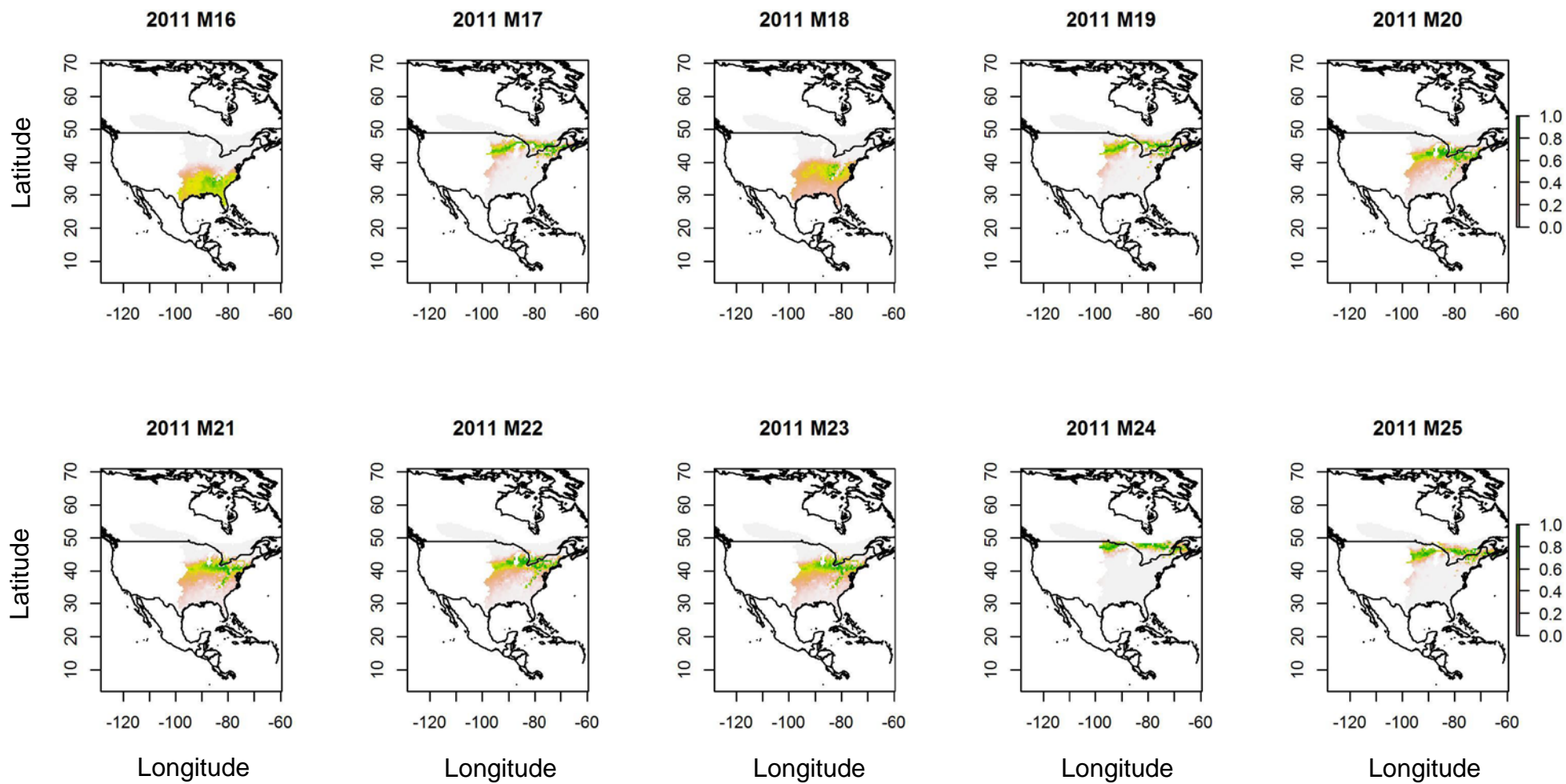

Supplement: Supplementary file 6 — Natal population assignment probability surfaces for each sampled male, hatch-year ruby-throated hummingbird captured in 2011. Feathers were collected in Fort Morgan, Alabama, USA during autumn. (PDF 909 kb) [file 40462_2017_120_MOESM6_ESM.pdf]

2014 F1

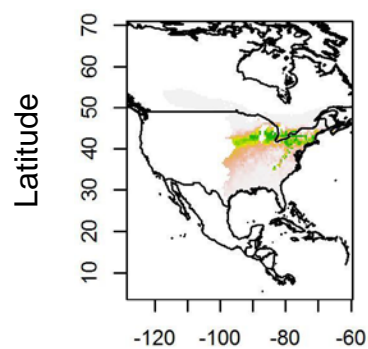

2014 F2

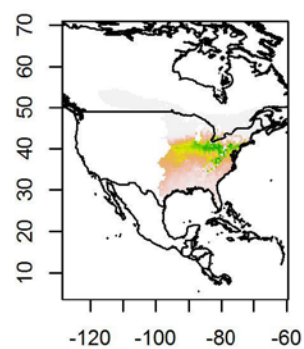

2014 F3

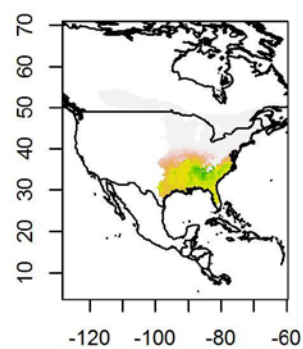

2014 F4

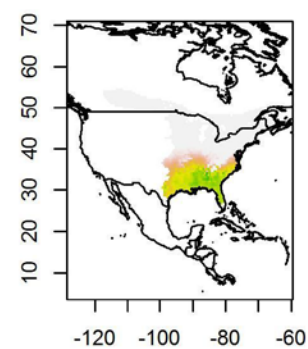

2014 F5

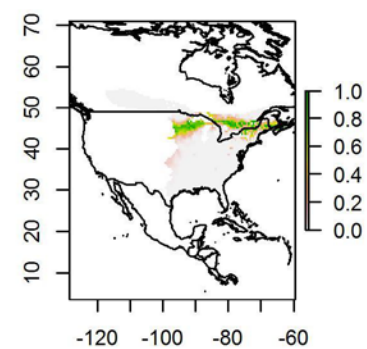

2014 F6

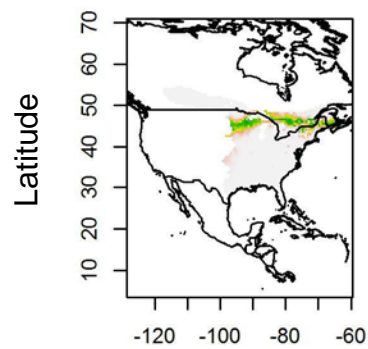

2014 F7

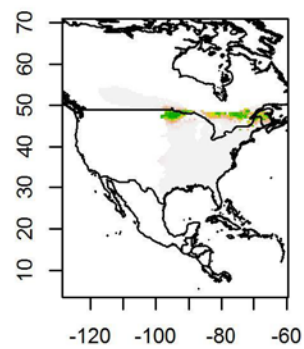

2014 F8

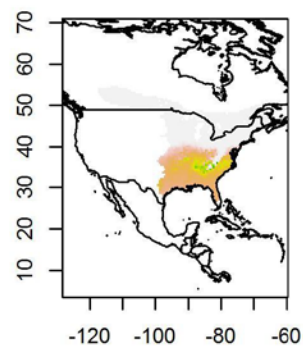

2014 F9

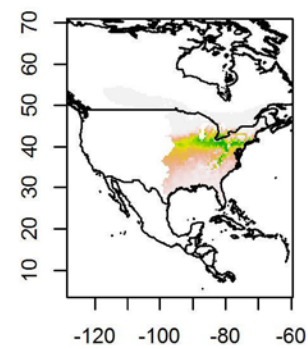

2014 F10

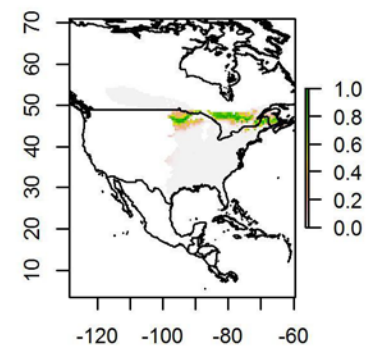

2014 F11

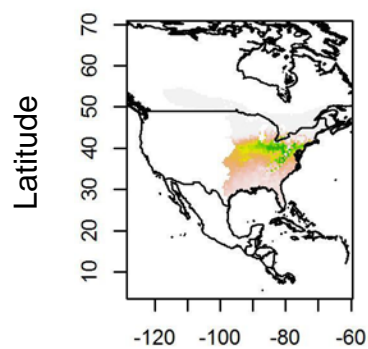

2014 F12

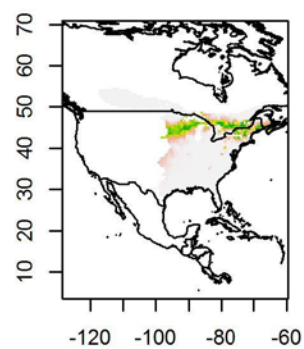

2014 F13

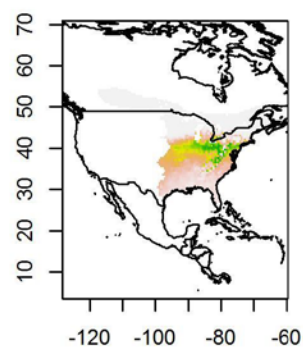

2014 F14

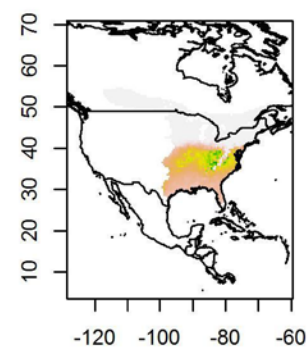

2014 F15

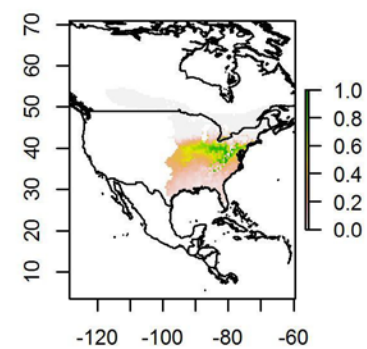

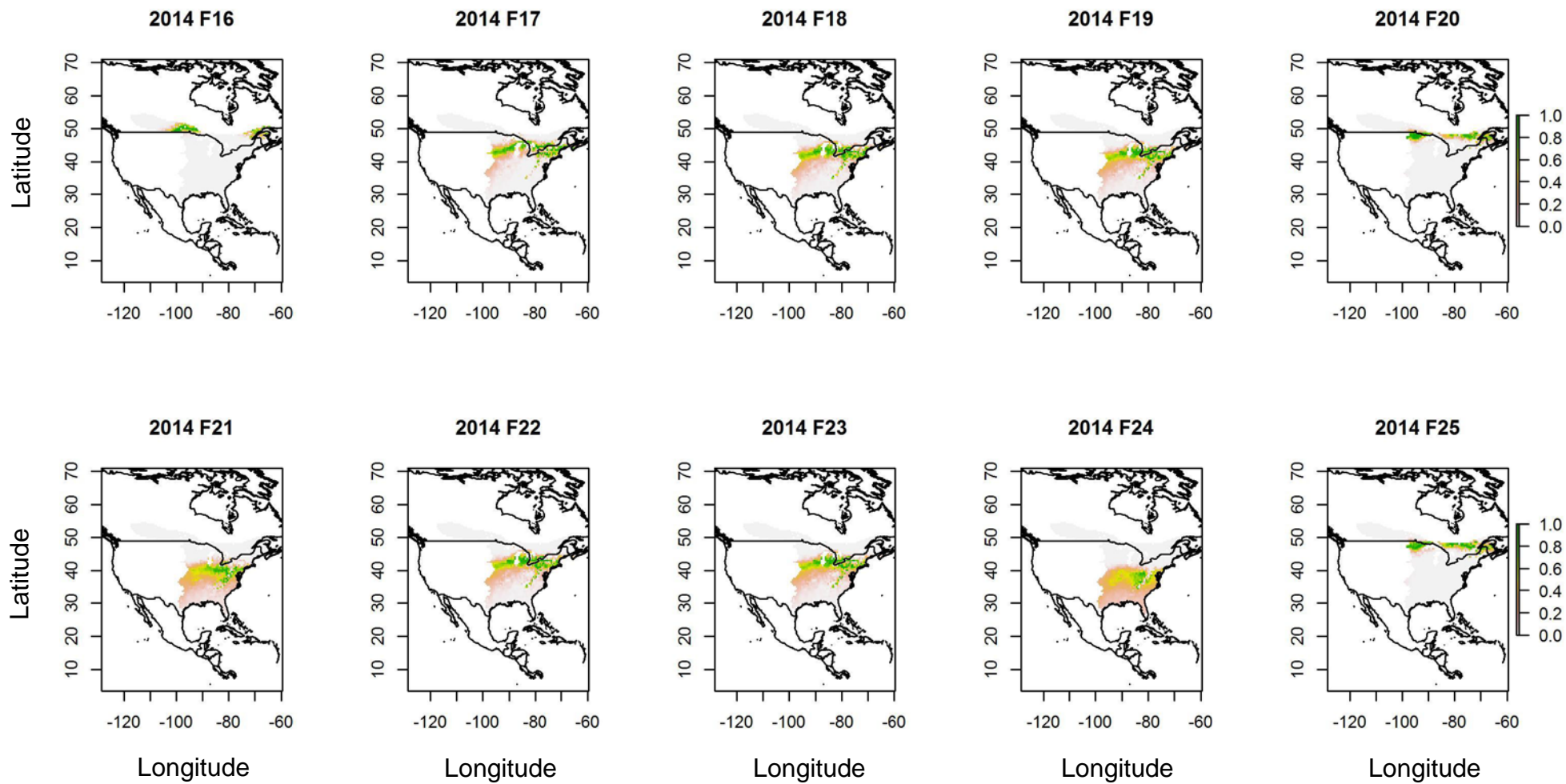

Supplement: Supplementary file 7 — Natal population assignment probability surfaces for each sampled female, hatch-year ruby-throated hummingbird captured in 2014. Feathers were collected in Fort Morgan, Alabama, USA during autumn. (PDF 906 kb) [file 40462_2017_120_MOESM7_ESM.pdf]

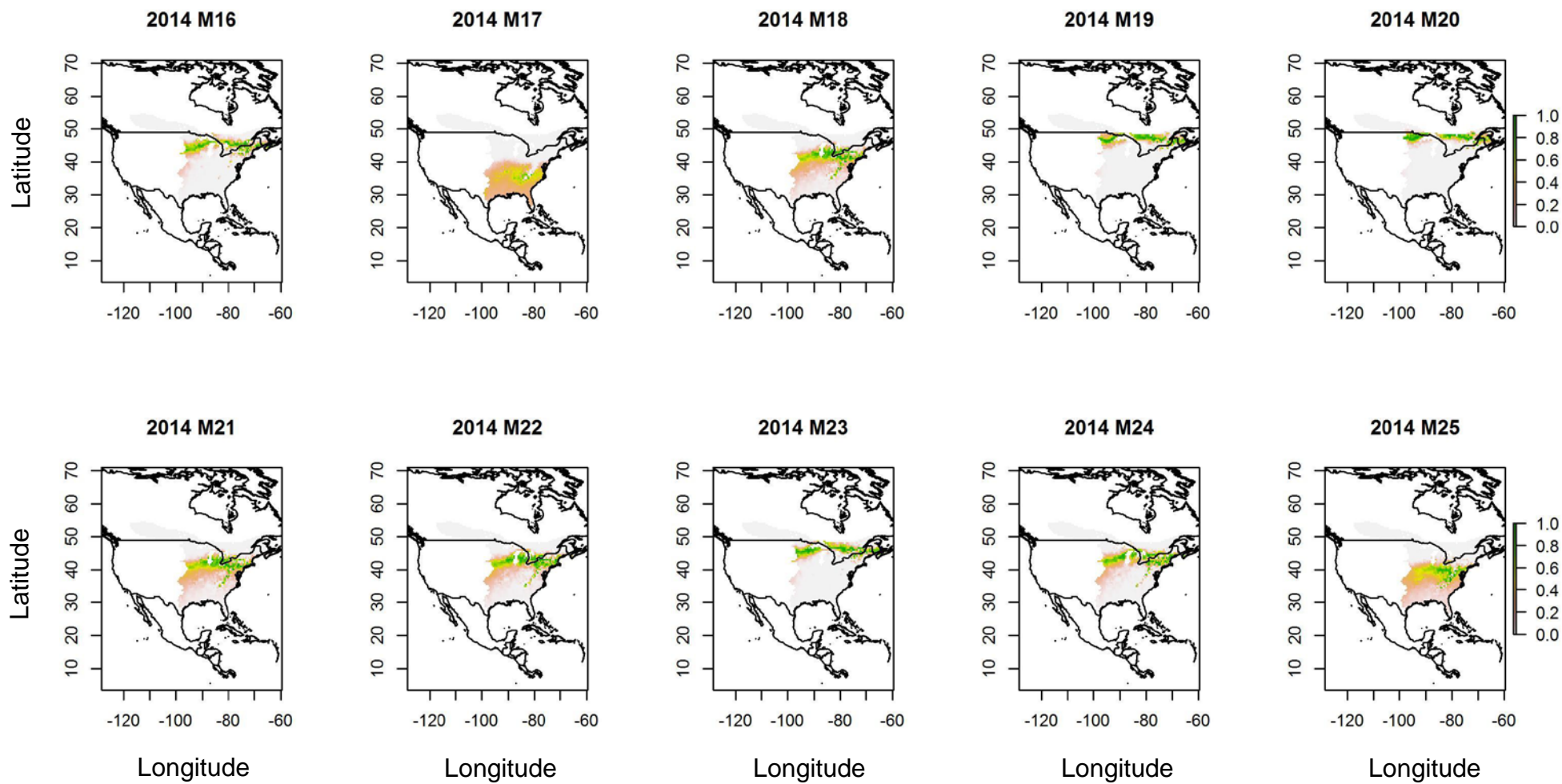

Supplement: Supplementary file 8 — Natal population assignment probability surfaces for each sampled male, hatch-year ruby-throated hummingbird captured in 2014. Feathers were collected in Fort Morgan, Alabama, USA during autumn. (PDF 752 kb) [file 40462_2017_120_MOESM8_ESM.pdf]
